# Mediating Role of Entrepreneurial Self-Efficacy and Prosocial Tendency in the Relation Between College Students’ Post-traumatic Growth and Entrepreneurial Intention in the Post-COVID-19 Era

**DOI:** 10.3389/fpsyg.2022.861484

**Published:** 2022-04-07

**Authors:** Lingjie Wang, Jianhao Huang

**Affiliations:** ^1^Hengshui University, Hengshui, China; ^2^Dhurakij Pundit University, Bangkok, Thailand

**Keywords:** the post-COVID-19 era, post-traumatic growth, entrepreneurial intention, entrepreneurial self-efficacy, prosocial tendency

## Abstract

In this study, we explore the psychological mechanisms underlying the relation between college students’ post-traumatic growth and their entrepreneurial intentions in the post-COVID-19 era. Using the post-traumatic growth, entrepreneurial self-efficacy, prosocial tendency, and entrepreneurial intention scales, we tested 690 valid samples of Chinese undergraduates (including 445 men and 245 women). The results revealed that post-traumatic growth of college students in the post-COVID-19 era will have a significant and positive effect on their entrepreneurial intentions. Additionally, the results indicated that students’ entrepreneurial self-efficacy and prosocial tendencies play a partial mediation role between post-traumatic growth and entrepreneurial intentions in the post-COVID-19 era and that there is a chain mediating effect between students’ entrepreneurial self-efficacy and prosocial tendencies. This study provides valuable insights into the influence of post-traumatic growth on entrepreneurial intentions among college students in the post-COVID-19 era and suggests that colleges and universities can improve students’ entrepreneurial intentions by adopting measures to foster their post-traumatic growth, entrepreneurial self-efficacy, and prosocial tendencies.

## Introduction

The COVID-19 pandemic has plunged the global economy into a deep recession (Chen et al., 2020). Accordingly, several countries have prioritized economic recovery in the post-COVID-19 era (Skidelsky, 2020). Encouraging people to start their businesses can significantly increase effective supply, bring vitality to our economy, hasten the development of new industries, increase employment and residents’ income, and promote economic and social development (Valliere and Peterson, 2009). However, enhancing people’s entrepreneurial intentions is crucial to help them build up their businesses (Kusumawijaya, 2020). Considering that university students are the new force for country’s development, exploring means to enhance college students’ entrepreneurial intentions in the post-COVID-19 era is crucial for the country’s economic recovery.

Factors such as an individual’s upbringing and growth experiences influence entrepreneurial intentions (Bird, 1988). An individual who experiences trauma, especially encountering health problems, has stronger entrepreneurial intentions (Williams and Shepherd, 2016). Resource theory states that people view a catastrophe as an opportunity to integrate resources when their resources have been destroyed after a disaster and engage in entrepreneurial activities following the catastrophic event (Shepherd and Patzelt, 2017). Traumatic experiences positively affect people’s psychology; in other words, they achieve post-traumatic growth (Tedeschi and Calhoun, 1995). The COVID-19 pandemic can be considered a growth environment and experience. Considering the aforementioned fact, the level of college students’ post-traumatic growth may affect their entrepreneurial intentions after their experience of the trauma caused by the pandemic.

The relation between post-traumatic growth and entrepreneurial intention and their underlying mechanisms have been ambiguous to date. Moreover, most researchers have considered post-traumatic growth as a dependent variable and have explored its influencing factors (An et al., 2017; Ersahin, 2020; Khursheed and Shahnawaz, 2020). A few researchers have treated post-traumatic growth as an independent variable to explore its effects on individual’s behavioral intentions (Boerner et al., 2017; Zeng et al., 2021). In the present study, we consider post-traumatic growth in the post-COVID-19 era as an independent variable to explore its effects on college students’ entrepreneurial intentions and its underlying mechanism of actions. By doing so, we aim to provide a reference for colleges to enhance college students’ entrepreneurial intentions and encourage them to build up their businesses, thereby bringing vitality to economic recovery in the post-COVID-19 era.

## Literature Review

### Post-traumatic Growth and Entrepreneurial Intention

Entrepreneurial intention is an individual’s belief to set up a new venture and consciously plan to put it into practice at a certain time in the future (Thompson, 2009). However, college students can build up their businesses only by enhancing their entrepreneurial intention (Li et al., 2021a). Factors such as personal experience, environment, cognition, and demographic factors affect entrepreneurial intentions; among these factors, researchers have exclusively focused on personal experience and external environment (Bilgiseven and Kasmolu, 2019). As an environment and experience for individuals, post-traumatic growth caused by the COVID-19 pandemic may be closely related to college students’ entrepreneurial intentions.

Post-traumatic growth (PTG) refers to the positive changes in individuals’ psychology experienced after struggling with a traumatic event (Tedeschi and Calhoun, 1995). A typical traumatic event such as the COVID-19 pandemic poses a threat to physical and mental health of Chinese college students (Zeng et al., 2021); thus, students may experience varying degrees of PTG in the post-COVID-19 era. Individuals who experience PTG can more positively meet the challenges of traumatic events and come up with innovative ideas (Han et al., 2019). First, in terms of entrepreneurial traits, individuals who experience trauma, especially those who have encountered health problems, have stronger entrepreneurial intentions (Williams and Shepherd, 2016). Second, in terms of entrepreneurial resources, according to resource theory, as people’s resources have been destroyed after a disaster, they view a catastrophe as an opportunity to integrate resources, and thus, they are motivated to build up their businesses following the catastrophic event (Shepherd and Patzelt, 2017). Moreover, studies on the theory and conceptual model of PTG have indicated that individuals who have achieved PTG possess the following characteristics: enhanced self-confidence, dynamic personality, improved personal relationships, optimistic mindset, and a stronger sense of responsibility (Tedeschi and Calhoun, 1996; Davis et al., 1998). These qualities are essential for entrepreneurs and positively influence the formation of entrepreneurial intentions (Prodan and Drnovsek, 2010). Therefore, we propose the following hypothesis (H1):

*H1*: College students’ PTG significantly and positively affects entrepreneurial intentions.

### Post-traumatic Growth, Entrepreneurial Self-Efficacy, and Entrepreneurial Intention

Bandura (1977) introduced the concept of self-efficacy, which has attracted widespread attention in the psychology circle, and defined self-efficacy as the individuals’ confidence in their ability to use their skills to perform an activity. Empirical studies have shown that self-efficacy can influence individuals’ performance, work practices, and work attitudes (Liu, 2019; Cepale et al., 2021). In the field of education, empirical studies have shown that teachers’ self-efficacy (e.g., classroom management efficacy, and teaching efficacy) can predict their job burnout and job satisfaction (Aloe et al., 2014; Perera et al., 2021), whereas college students’ self-efficacy (e.g., academic self-efficacy, career decision-making self-efficacy, and social self-efficacy) can predict their academic performance, career choice commitment, and psychological wellbeing (Chemers et al., 2001; Wang et al., 2006; Hong et al., 2021). By introducing self-efficacy to the field of entrepreneurship, Boyd and Vozikis (1994) developed the concept of entrepreneurial self-efficacy, namely, the level of an individual’s belief that they can successfully assume various entrepreneurial roles and complete various entrepreneurial tasks. Entrepreneurial self-efficacy is an important prerequisite for potential entrepreneurs to start their business (Jakopec et al., 2013).

Numerous studies have revealed that PTG and self-efficacy are closely related and that the two affect each other. An individual with high self-efficacy can achieve PTG more quickly following a traumatic event. Thereafter, they will be mentally stronger and demonstrate greater self-efficacy (Robles-Bello et al., 2020; Kwak et al., 2021). In addition, PTG leads to positive changes in an individual’s cognition in many aspects, especially an increase in their inner strength (Mohd Shariff et al., 2021). The change enables them to feel more confident in themselves and demonstrate a high sense of self-efficacy under stressful events (Jia et al., 2017), thereby enhancing their entrepreneurial self-efficacy (Udayanan, 2019). Therefore, an individual who has experienced a trauma caused by the COVID-19 pandemic and has achieved PTG will be mentally strong and demonstrate a higher sense of entrepreneurial self-efficacy.

Self-efficacy theory states that people are motivated to make a certain move and can overcome potential difficulties only if they believe that they can accomplish it (Bandura, 1999). Numerous empirical studies have revealed that entrepreneurial self-efficacy is a crucial predictive variable of entrepreneurial intention (Wu et al., 2019; Li et al., 2020; Zhang et al., 2021). Furthermore, entrepreneurial self-efficacy significantly influences other behaviors such as entrepreneurial performance and entrepreneurial intention (Elnadi and Gheith, 2021). Therefore, entrepreneurial self-efficacy, a key explanatory variable in the formation of an individual’s entrepreneurial intentions, is vital for entrepreneurs to seize opportunities, organize resources, establish a company, and achieve success (Tantawy et al., 2021). Taken together, an individual with a strong sense of entrepreneurial self-efficacy will be confident enough to cope with the difficulties encountered in the entrepreneurial process, which can finally enhance their entrepreneurial intentions.

In conclusion, in the post-COVID-19 era, college students who achieve PTG will be mentally stronger and demonstrate a stronger sense of self-efficacy, which make them believe that they can grasp entrepreneurial opportunities and address entrepreneurial risks with ease, thereby displaying will to build up their businesses. Some empirical studies have revealed that entrepreneurial self-efficacy directly affects entrepreneurial intention. Moreover, entrepreneurial self-efficacy has a mediating effect on entrepreneurial intention (Wenqing et al., 2019; Elnadi and Gheith, 2021). Therefore, we propose the following hypothesis (H2):

*H2*: College students’ entrepreneurial self-efficacy plays a mediating role in the effects of PTG on entrepreneurial intention.

### Post-traumatic Growth, Prosocial Tendency, and Entrepreneurial Intention

Prosocial tendency, based on altruism, is the willingness to involve in activities that benefit other people (Carlo et al., 2003). According to a study, the COVID-19 pandemic has encouraged people to engage in prosocial activities (Sin et al., 2021). This is because people’s mindsets have changed, and they have achieved PTG following the pandemic (Singla et al., 2021). In addition, they have experienced positive changes in their attitudes toward personal relationships, worldview, and life values. Such positive changes further motivate them to be more willing to help and cooperate with others, as well as more actively participate in public welfare activities (Vollhardt, 2009). Empirical studies have found that individuals with a history of trauma display stronger prosocial tendencies than those without any history of trauma (McGinley et al., 2009). Therefore, college students, influenced by PTG, may show greater prosocial tendencies after experiencing trauma due to the COVID-19 pandemic.

Kim et al. (2020) examined 179 Korean entrepreneurs who experienced traumatic events to explore the psychological causes that compelled them to start their businesses. The authors observed that prosocial tendency promoted entrepreneurial intention. This is because individuals who have suffered trauma have a strong desire to help others, and the desire motivates individuals to solve other people’s difficulties by starting businesses (Le et al., 2020). Previous studies have documented that entrepreneurs’ prosocial intentions contribute to entrepreneurial intention and opportunity identification (Al-Harasi et al., 2021). The prosocial tendency can predict entrepreneurial intentions (Yu et al., 2020). Prosocial tendencies are required for engaging in entrepreneurial activities, and individuals engaging in entrepreneurial activities have a strong prosocial inclination (Douglas and Prentice, 2019). Therefore, college students with prosocial tendencies may have firmer entrepreneurial intention.

Empirical studies have shown that the prosocial tendency can play a mediating role in empathy forecasting entrepreneurial intention (Tiwari et al., 2020), and it has also demonstrated mediation effects in other empirical studies (Zhu and Akhtar, 2014; Carlo et al., 2016). Altogether, the college students who have undergone coronavirus-induced traumatic events may develop a positive attitude toward interpersonal relationships, display greater prosocial tendencies, and thus have a strong desire to start their businesses (Le et al., 2020). Therefore, we propose the following hypothesis (H3):

*H3*: College students’ prosocial tendencies play a mediating role in the effects of PTG on entrepreneurial intentions.

### Post-traumatic Growth, Entrepreneurial Self-Efficacy, Prosocial Tendencies, and Entrepreneurial Intentions

Individuals who experience PTG demonstrate a strong entrepreneurial self-efficacy, and individuals with high self-efficacy are more likely to exhibit prosocial tendencies such as comforting, sharing with, and helping others (De Caroli and Sagone, 2013). A study by Liu and Ngai (2019) found that self-efficacy can influence individuals’ prosocial tendencies. This is because individuals with a high sense of self-efficacy exhibit enough confidence in social activities. Additionally, they feel that they are capable of handling problems in their own way, consider the entire society or organization, and take more responsibility for the society or organization, thus displaying stronger prosocial tendency (Davis et al., 2021), and showing the willingness to start their businesses while fulfilling their social responsibilities (Douglas and Prentice, 2019).

Social cognitive theory suggests an interaction between environmental factors, individual factors, and individual behavior (Bandura’s, 1977), implying that both environmental and individual factors influence individual behavior. According to the theory, environmental factors, as resources for individuals to enhance their self-prediction and effortful control, provide them with precise information that influences the direction and intensity of their behavior, while the process by which environmental factors influence behavior varies depending on the individual’s cognitive features and levels (Schunk and DiBenedetto, 2020). Clearly, environmental and individual factors contribute equally in influencing individual behavior. Researchers have explored college students’ entrepreneurial intentions according to the social cognitive theory, while considering the COVID-19 pandemic or social support as an environmental factor and entrepreneurial self-efficacy as an individual factor (Neneh, 2020; Zhang and Huang, 2021). In the present study, we considered PTG in the post-COVID-19 era as an environmental factor, entrepreneurial self-efficacy and prosocial tendency as individual factors, and entrepreneurial intention as a behavioral intention to explore the direct and indirect effects of PTG, entrepreneurial self-efficacy, and prosocial tendencies on entrepreneurial intentions in the post-COVID-19 era. To summarize, the social cognitive theory may also be used to explore college students’ entrepreneurial intentions. Specifically, PTG is likely to enhance entrepreneurial self-efficacy and prosocial tendencies of college students in the post-COVID-19 era, whereas college students’ entrepreneurial self-efficacy may also enhance prosocial tendencies, thus improving their entrepreneurial intentions. Therefore, we propose the following hypothesis (H4):

*H4*: College students’ entrepreneurial self-efficacy and prosocial tendencies have a chain mediating effect on the influence of PTG on entrepreneurial intention.

## Research Method

### Research Participants

College students from a university in Hebei Province, China, were included in the study. The university is a truly representative sample because the Hebei Province is hard hit by the COVID-19 pandemic. Moreover, the university is a model school of entrepreneurship education with a good entrepreneurial atmosphere for college students. Using convenience sampling, we distributed 750 questionnaires. After eliminating 60 invalid questionnaires, 690 questionnaires were considered valid (including those of 445 males and 245 females). This research was conducted in accordance with the Declaration of Helsinki, and all participants’ privacy, feelings, and intentions were fully considered. Participants voluntarily filled in the questionnaire and signed an informed consent (Goodyear et al., 2007).

### Analytical Strategy

The analysis method of this study included the pilot test and formal stages. Furthermore, 138 valid questionnaires were returned in the pilot test stage. To test the reliability and validity of the scale, we performed an exploratory factor analysis and an reliability analysis of this part of the data by using SPSS 21.0. In the formal stage, 690 valid questionnaires were received. We performed descriptive statistics and correlation analysis of this part of the data by using SPSS 21.0 and tested the measurement model and structural model by using AMOS 21.0.

### Research Instruments

Owing to the large sample size of this study, $x^{2}$ increased while performing a confirmatory factor analysis of the scale; thus, other adaptation indicators were considered (Hu and Be Ntler, 1998). The results revealed that all other fit indices met the standards established by Hsiao et al. (2015): RMR < 0.08, GFI > 0.8, AGFI >0.8, PNFI >0.5, PGFI >0.5, NFI > 0.8, IFI > 0.8, CFI > 0.8, SRMR <0.08, and RMSEA <0.08, indicating a good fit of the measurement model for each scale.

#### Post-traumatic Growth

In this study, we defined PTG as the positive psychological changes of college students after experiencing traumatic events. According to Tedeschi and Calhoun (1996) measure of PTG, we added the word “the COVID-19 pandemic” to the beginning of each item and established a PTG scale for the post-COVID-19 era. The scale comprised 13 items that were divided into four dimensions: relating to others, new possibilities, personal strength, and spiritual change. The participants were asked to rate the level of their PTG on a 6-point Likert scale (1 = not at all and 6 = very much). The higher the score, the higher was the level of PTG. An exploratory factor analysis revealed that factor loadings ranged from 0.408 to 0.828, the explanation rate of the scale was 68.451%. A reliability analysis revealed that Cronbach’s α for each dimension of the scale ranged from 0.783 to 0.857, Cronbach’α for the overall scale was 0.936. Furthermore, a confirmatory factor analysis revealed that $x^{2}$ = 752.686, df = 59, $x^{2}/df\;$= 12.757, RMR = 0.089, GFI = 0.86, PNFI = 0.639, PGFI = 0.558, CFI = 0.855, IFI = 0.856, and SRMR = 0.074.

#### Entrepreneurial Self-Efficacy

In this study, entrepreneurial self-efficacy was defined as college students’ confidence or belief in their ability to start their businesses by judging and assessing their own ability to achieve certain entrepreneurial behaviors. According to Barbosa et al. (2007) measure of entrepreneurial self-efficacy, we established an entrepreneurial self-efficacy scale comprising four dimensions and 15 items: tolerance ambiguity self-efficacy, opportunity-identification self-efficacy, relationship self-efficacy, and managerial self-efficacy. The participants were asked to rate the level of their entrepreneurial self-efficacy on a 5-point Likert scale (1 = not at all and 5 = fully); the higher the score, the higher was the level of entrepreneurial self-efficacy. An exploratory factor analysis revealed that factor loadings ranged from 0.421 to 0.831, the explanation rate of the scale was 73.289%. A reliability analysis revealed that Cronbach’s α for each dimension of the scale ranged from 0.776 to 0.890, Cronbach’α for the overall scale was 0.935. The confirmatory factor analysis revealed that $x^{2}$= 741.188, df= 84, $x^{2}∕df$ = 8.824, RMR = 0.039, GFI = 0.878, AGFI = 0.826, PNFI = 0.713, PGFI = 0.615, NFI = 0.891, IFI = 0.902, CFI = 0.902, SRMR = 0.0536, and RMSEA = 0.107.

#### Prosocial Tendencies

In this study, we defined prosocial tendencies as friendly and positive tendencies exhibited by people in social intercourse. We used the Prosocial tendency Scale revised by Kou et al. (2007), which was applied in measuring prosocial tendencies of Chinese adolescents. The scale comprised six dimensions and 26 items: public, emotional, altruism, compliant, anonymity, and dire. The participants were asked to rate the level of their prosocial tendencies on a 5-point Likert scale (1 = not at all and 5 = fully). The higher the score, the more pronounced was their prosocial tendencies. An exploratory factor analysis revealed that factor loadings ranged from 0.441 to 0.851, the explanation rate of the scale was 69.91%. A reliability analysis revealed that Cronbach’s α for each dimension of the scale ranged from 0.809 to 0.895, Cronbach’α for the overall scale was 0.940. The confirmatory factor analysis revealed that $x^{2}\;$ = 1931.72, df = 237, $x^{2}/df$ = 8.151, RMR = 0.046, GFI = 0.824, PNFI = 0.706, PGFI = 0.651, CFI = 0.902, SRMR = 0.065, and RMSEA = 0.102.

#### Entrepreneurial Intention

In this study, we defined entrepreneurial intention as a psychological state that directs an entrepreneur’s attention, energy, and behaviors toward a specific goal. According to Li et al. (2021b) measure of entrepreneurial intention, we established an entrepreneurial intention scale comprising two dimensions (namely, goal intentions and implementation intentions) and 10 items. The participants were asked to rate the level of their entrepreneurial intention on a 7-point Likert scale (1 = not at all and 7 = fully). The higher the score, the higher was the level of entrepreneurial intention. An exploratory factor analysis revealed that factor loadings ranged from 0.490 to 0.841, the explanation rate of the scale was 76.014%. A reliability analysis revealed that Cronbach’s α for each dimension of the scale ranged from 0.928 to 0.936, Cronbach’α for the overall scale was 0.959. A confirmatory factor analysis revealed that $x^{2}$ = 548.252, df = 34, $x^{2}∕df$ = 16.125, RMR = 0.09, GFI = 0.864, PNFI = 0.690, PGFI = 0.534, NFI = 0.913, IFI = 0.918, CFI = 0.918, and SRMR = 0.042.

### Model Comparison of CFA

To explore whether higher common factors exist in the measurement models, we compared three models in this study, namely, Model 1 (single first-order factor model), Model 2 (16 first-order factors model), and Model 3 (4 s-order factors model). As shown in Table 1, Model 2 was significantly different from Model 1 (△ $x^{2}$ = 8492.72, △ df = 120, *p* < 0.001), Model 3 was significantly different from Model 1 (△$x^{2}$ = 7706.5, △df = 22, *p* < 0.001), and Model 2 was significantly different from Model 3 (△$x^{2}$ = 786.22, △df = 98, *p* < 0.001). According to the fitness index, $x^{2}$, RMR, GFI, CFI, and PNFI in Model 2 were superior to those in the other models, indicating that Model 2 was the excellent model among the three models in terms of fitness and that no higher common factors were present in the measurement models.

**Table 1:** Model comparison of CFA.

Model	*х^2^*	*df*	RMR	GFI	CFI	PNFI	**△** *х^2^*	**△** *df*	*p*
Model 1	21394.05	1829	0.09	0.46	0.49	0.45	–	–	–
Model 2	12901.33	1709	0.06	0.66	0.71	0.63	–	–	–
Model 3	13687.55	1807	0.07	0.64	0.69	0.61	–	–	–
M2–M1	–	–	–	–	–	–	8492.72	120	0.000
M3–M1	–	–	–	–	–	–	7706.5	22	0.000
M2–M3	–	–	–	–	–	–	786.22	98	0.000

*Model 1, Single first-order factor model; Model 2, 16 first-order factors model, and Model 3, 4 second-order factors model*.

## Study Results

### Descriptive Statistics and Correlation Analysis

Table 2 presents the correlation between descriptive statistics data and Pearson correlation coefficients for all observable variables. The results revealed a significant positive correlation (*p* < 0.01) between two of each observable variable. Correlation coefficients ranged from 0.278 to 0.809, indicating no serious collinearity problem and satisfying the requirements of the structural equation model hypothesis testing.

**Table 2:** Descriptive statistics and correlation analysis between the observable variables.

Variables	M	SD	1	2	3	4	5	6	7	8	9	10	11	12	13	14	15	16
1	3.61	0.99	1															
2	3.84	0.94	0.564^***^	1														
3	4.13	0.82	0.524^***^	0.655^***^	1													
4	4.27	0.93	0.396^***^	0.648^***^	0.642^***^	1												
5	3.54	0.69	0.416^***^	0.614^***^	0.585^***^	0.464^***^	1											
6	3.35	0.75	0.494^***^	0.621^***^	0.577^***^	0.524^***^	0.687^***^	1										
7	3.64	0.68	0.457^***^	0.512^***^	0.541^***^	0.485^***^	0.672^***^	0.646^***^	1									
8	3.08	0.80	0.398^***^	0.505^***^	0.446^***^	0.392^***^	0.605^***^	0.649^***^	0.676^***^	1								
9	3.48	0.73	0.374^***^	0.343^***^	0.456^***^	0.397^***^	0.429^***^	0.430^***^	0.470^***^	0.477^***^	1							
10	3.76	0.78	0.382^***^	0.476^***^	0.500^***^	0.440^***^	0.432^***^	0.422^***^	0.395^***^	0.278^***^	0.299^***^	1						
11	4.00	0.75	0.415^***^	0.479^***^	0.516^***^	0.440^***^	0.514^***^	0.510^***^	0.529^***^	0.392^***^	0.306^***^	0.666^***^	1					
12	3.67	0.61	0.427^***^	0.483^***^	0.438^***^	0.437^***^	0.459^***^	0.475^***^	0.569^***^	0.441^***^	0.466^***^	0.470^***^	0.549^***^	1				
13	3.67	0.74	0.443^***^	0.482^***^	0.442^***^	0.435^***^	0.564^***^	0.474^***^	0.458^***^	0.486^***^	0.477^***^	0.422^***^	0.584^***^	0.576^***^	1			
14	3.91	0.67	0.412^***^	0.505^***^	0.375^***^	0.395^***^	0.507^***^	0.462^***^	0.453^***^	0.336^***^	0.421^***^	0.530^***^	0.578^***^	0.548^***^	0.587^***^	1		
15	4.61	1.33	0.530^***^	0.608^***^	0.643^***^	0.528^***^	0.578^***^	0.647^***^	0.590^***^	0.646^***^	0.481^***^	0.517^***^	0.557^***^	0.519^***^	0.607^***^	0.500^***^	1	
16	4.30	1.14	0.504^***^	0.538^***^	0.631^***^	0.487^***^	0.549^***^	0.633^***^	0.562^***^	0.628^***^	0.468^***^	0.461^***^	0.493^***^	0.522^***^	0.607^***^	0.394^***^	0.809^***^	1

1, relating to others, 2, new possibilities, 3, personal strength, 4, spiritual change, 5, tolerance ambiguity self-efficacy, 6, opportunity-identification self-efficacy, 7, relationship self-efficacy, 8, managerial self-efficacy, 9, public, 10, anonymous, 11, altruism, 12 = compliant, 13, emotional, 14, dire, 15, goal intention, and 16, implementation intention. ^***^p < 0.001.

### Common Method Variance

In this study, Harman’s single-factor analysis was used to detect common method variance (CMV). The results revealed that the single-factor’s maximum explained amount of variation was 39.18%, indicating no serious CMV problem. For the rigor of the study, we further compared the single-factor model CFA with the multi-factor model CFA for adaptation. The results indicated that the multi-factor model $x^{2}$ was much lower than the single-factor model (*p* < 0.05), indicating that the adaptation of the multi-factor model was significantly better than that of the single-factor model, and the CMV problem in this study was not serious (see Table 3).

**Table 3:** Comparison between single-factor model and multi-factor model.

Model	*х^2^*	df	**△** *х^2^*	**△** *df*	*p*
Single-factor model	21394.047	1829	8492.713	120	0.001
Multi-factor model	12901.334	1709			

### Structural Model

**Figure 1:**
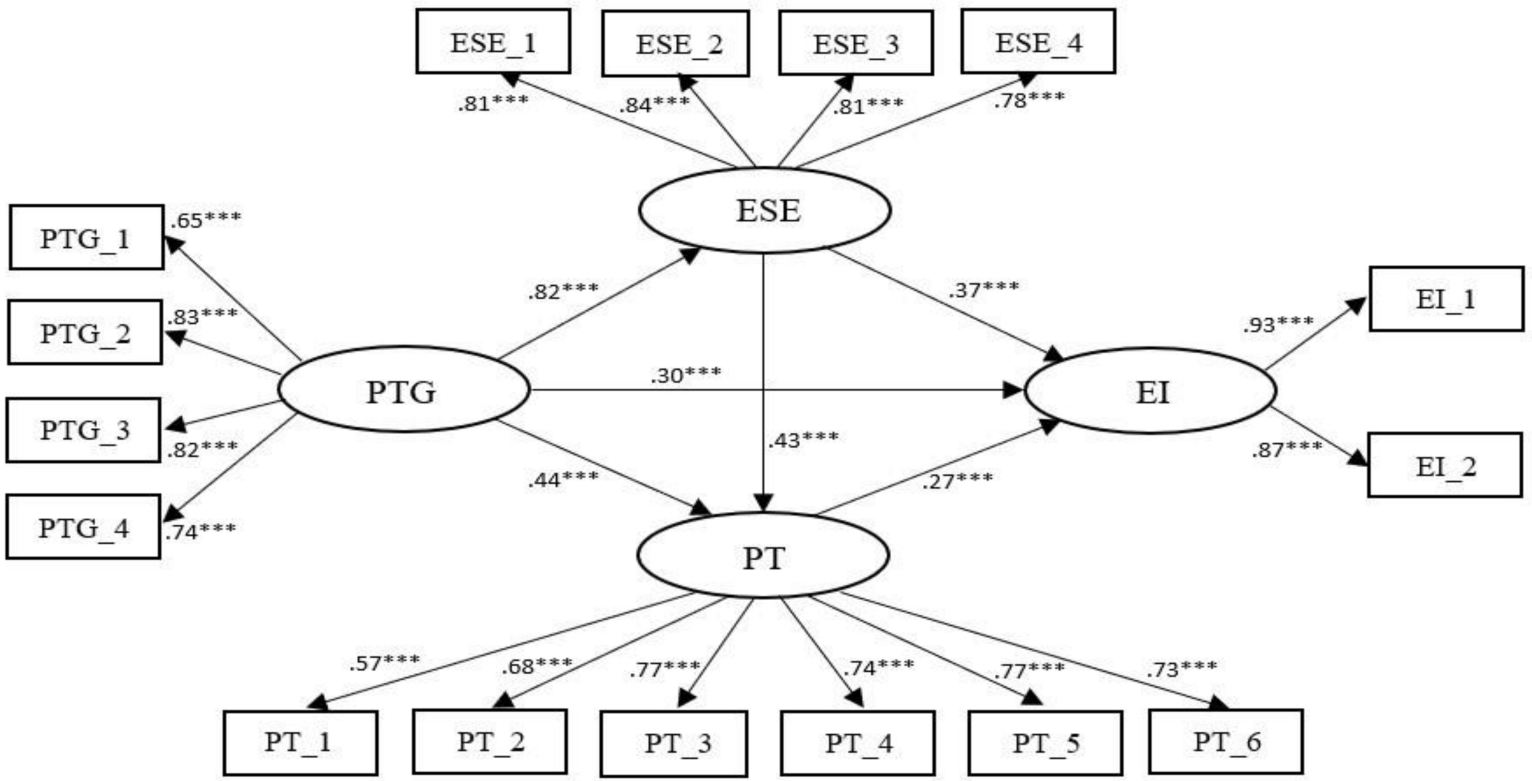
Standardized parameter estimation of the final model. PTG, Post-traumatic growth, ESE, Entrepreneurial self-efficacy, PT, Prosocial tendencies, and EI, Entrepreneurial intention; ^***^p < 0.001.

The model (see Figure 1) examined the relation among PTG, entrepreneurial self-efficacy, prosocial tendencies, and entrepreneurial intention. The result revealed that $x^{2}$ = 836.758, df = 98, and $x^{2}∕df$ = 8.538. Other adaptation indicators were to be considered because the large sample size of this study increased $x^{2}∕df$ ratio (Hu and Be Ntler, 1998). Furthermore, RMR = 0.032, GFI = 0.874, PGFI = 0.630, CFI = 0.9, IFI = 0.901, SRMR = 0.048, and RMSEA = 0.105, indicating that all fit indices reached acceptable levels (Hsiao et al., 2015). As a precautionary measure, this study further used the nonparametric percentile Bootstrap method to test mediation path effects. The results indicated that the 95% confidence intervals for the direct, indirect, and total effects of the bias-corrected nonparametric percentile did not contain 0 (see Table 4). Specifically, these results validated H1: the effect of PTG on entrepreneurial intention (*β* = 0.298, *p* < 0.01); H2: entrepreneurial self-efficacy partially mediates the effect of PTG on entrepreneurial intention (*β* = 0.302, *p* < 0.01); H3: the prosocial tendency partially mediates the effect of PTG on entrepreneurial intention (*β* = 0.117, *p* < 0.01); and H4: entrepreneurial self-efficacy and prosocial tendencies have a chain mediating effect in the relation between PTG and entrepreneurial intention (*β* = 0.095, *p* < 0.01).

**Table 4:** Summary table of path effects.

Path	Effect	95% LLCI	95% ULCI
Direct effect	0.298^**^	0.160	0.430
Indirect effect1	0.302^**^	0.198	0.422
Indirect effect2	0.117^**^	0.063	0.191
Indirect effect3	0.095^**^	0.052	0.148
Total effect	0.515^**^	0.403	0.635

Bootstrapping random sampling 5,000 times; direct effect = PTG→entrepreneurial intention; indirect effect 1 = PTG→*entrepreneurial self-efficacy*→entrepreneurial intention; indirect effect 2 = PTG→*prosocial tendencies*→entrepreneurial intention; and indirect effect 3 = PTG→*entrepreneurial self-efficacy*→*prosocial tendencies*→entrepreneurial intention. ^**^p < 0.010.

### Model Comparison of SEM

To illustrate that the final constructed chain mediation model outperforms other models in terms of fitness, we used SEM to construct four models (see Table 5) in this study, namely, model 1 (PTG → EI), model 2 (PTG → ESE → EI), model 3 (PTG → PT → EI), and model 4 (PTG → ESE → PT → EI). We compared the fitness of model 4 with that of other models, and the results are shown in Table 5. Model 4 was significantly different from model 1 (△ $x^{2}$ = 768.32, △df = 90, *p* < 0.001), Model 4 was significantly different from model 2 (△ $x^{2}\;$ = 595.03, △df = 66, *p* < 0.001), and Model 4 was significantly different from model 3 (△ $x^{2}$ = 400.82, △ df = 47, *p* < 0.001), indicating that the chain mediation model constructed finally in this research showed a significant difference with the fit measure of other models. In Model 4, the three variables, namely PTG, entrepreneurial self-efficacy, and prosocial tendencies, together explained 76% of the entrepreneurial intention (SMC = 0.76), and the interpretation ratios in Model 1, Model 2, and Model 3 were 66% (SMC = 0.66), 74% (SMC = 0.74), and 72% (SMC = 0.72), respectively, indicating that the final chain mediation model constructed in this study had the highest explanatory power for entrepreneurial intentions.

**Table 5:** Model comparison of SEM.

Model	*х^2^*	df	RMR	GFI	CFI	PNFI	****△** *х^2^***	**△** *df*	*p*
Model 1	68.436	8	0.03	0.97	0.98	0.52	–	–	–
Model 2	241.727	32	0.03	0.94	0.96	0.68	–	–	–
Model 3	435.939	51	0.03	0.91	0.92	0.71	–	–	–
Model 4	836.758	98	0.03	0.87	0.90	0.73	–	–	–
M4-M1	–	–	–	–	–	–	768.32	90	0.000
M4-M2	–	–	–	–	–	–	595.03	66	0.000
M4-M3	–	–	–	–	–	–	400.82	47	0.000

*PTG, Post-traumatic growth, ESE, Entrepreneurial self-efficacy, PT, Prosocial tendencies, and EI, Entrepreneurial intention. Model 1 = PTG→EI, Model 2 = PTG→ESE→EI, Model 3 = PTG→PT→EI, and Model 4 = PTG→ESE→PT→EI*.

## Discussion

The study results indicated that PTG significantly and positively affects entrepreneurial intention of Chinese college students who have experienced the trauma due to COVID-19 pandemic. The result is consistent with the findings of Williams and Shepherd (2016) indicating that the entrepreneurial intention of college students grows higher with an increase in the levels of their PTG. The reason is that the COVID-19 pandemic has affected college students’ physical and mental health to varying degrees, and college students undergo positive changes in their spiritual journey after coping with the traumatic event (Tedeschi and Calhoun, 1995). The changes enable college students to face the traumatic event and rise to life challenges with more positive emotions, eventually encouraging them to come up with creative ideas and enhancing their entrepreneurial intention. In addition, according to the theory of “altruism from suffering,” the positive growth after trauma encourages college students to help others in practical ways. College students who have experienced trauma are motivated to seize the opportunity of entrepreneurship to solve problems when they see other people traumatized by social problems.

The study results indicated that college students’ entrepreneurial self-efficacy partially mediates the relation between PTG and entrepreneurial intention, which is consistent with the previous findings that as the level of PTG increases, entrepreneurial self-efficacy increases; high levels of entrepreneurial self-efficacy are associated with increased entrepreneurial intention (Jia et al., 2017; Zhang and Huang, 2021). The reason is that after college students experience the trauma caused by the COVID-19 pandemic, their psychological quality becomes stronger, and they exhibit greater tolerance to stress and become confident and optimistic, which enhance their entrepreneurial self-efficacy. Individuals with a strong sense of entrepreneurial self-efficacy hold a stronger belief that they can successfully start their own businesses in an uncertain environment, thus displaying stronger entrepreneurial intention. College students hold a specific belief about their active ability and acquire the power of positive thinking after a traumatic event; thus, based on judgments of their abilities, knowledge, and experience, they plan to start their businesses. Entrepreneurial self-efficacy is the beginning of entrepreneurial intention and the key to stimulating entrepreneurial intention. Therefore, once college students aim to start their businesses, they should consciously cultivate their entrepreneurial self-efficacy and aim to achieve entrepreneurial goals with strong entrepreneurial intentions.

The findings confirmed that prosocial tendencies partially mediate the relation between PTG and entrepreneurial intention, further validating the findings of a previous study that with an increase in the level of an individual’s PTG, prosocial tendencies, and entrepreneurial intention of the individual increase (Al-Harasi et al., 2021; Sin et al., 2021). The reason is that after college students experience the trauma caused by the COVID-19 pandemic, they experience positive changes in their spiritual journey and recover from the trauma. Thus, they exhibit more prosocial tendencies, such as being obliging and caring for others, and display altruistic behaviors by helping others overcome the trauma. Thus, college students try to help traumatized people through prosocial tendencies, and this behavior prompts such students to transform into social entrepreneurs. On the one hand, the COVID-19 pandemic may inflict bodily and emotional injury to college students directly, for instance, being quarantined or seeing a friend or relative be diagnosed cast a shadow over them. On the other hand, they may not be traumatized, but they will be stimulated to provide help to others and society when they witness others’ injury or death caused by the infection. In doing so, they change their original perception of life and living, encouraging them to value life and time more. Therefore, they will be determined to challenge themselves to do something they wanted to do before but did not have the courage, such as starting a business.

This study determined that entrepreneurial self-efficacy and prosocial tendencies have a chain mediating effect on the relation between PTG and entrepreneurial intention, validating previous study findings. PTG affects entrepreneurial self-efficacy, and individuals with high entrepreneurial self-efficacy exhibit more prosocial tendencies, thereby promoting entrepreneurship. The reason is that after coping with trauma caused by the COVID-19 pandemic, college students’ psychological quality becomes stronger. They become highly confident of their ability to deal with problems and meet challenges, thereby exhibiting increased entrepreneurial self-efficacy. With an increase in the entrepreneurial self-efficacy, college students display more prosocial tendencies such as donating and volunteering. When helping other traumatized people, they realize that life is valuable and time is important, thus cherishing life and time more and realizing their life value by engaging in challenging activities such as starting businesses.

## Theoretical Contributions

The present research results make theoretical contributions to the literature on college students’ entrepreneurial intentions to some degree. The study has three major findings. First, PTG significantly and positively influences college students’ entrepreneurial intentions in the post-COVID-19 era. Second, both entrepreneurial self-efficacy and prosocial tendencies partially mediate the relationship between PTG and entrepreneurial intentions. Third, entrepreneurial self-efficacy and prosocial tendencies have a chain mediating effect between PTG and entrepreneurial intentions. Some studies have shown that individuals’ traumatic experiences can influence their entrepreneurial intentions (Williams and Shepherd, 2016; Shepherd and Patzelt, 2017); however, the impact of the COVID-19 pandemic on entrepreneurial intentions and its underlying mechanisms of action remain unclear. Furthermore, although previous studies have explored entrepreneurial intentions based on the social cognitive theory (Sweida and Reichard, 2013; Zhang and Huang, 2021), only a few of these studies perceived PTG as an environmental factor to explore entrepreneurial intentions in the post-COVID-19 era. Therefore, the influence of college students’ PTG on entrepreneurial intention and its underlying mechanisms of action should be further explored. The contributions of this study is the findings that college students’ PTG positively influences entrepreneurial intention in the post-COVID-19 era and that entrepreneurial self-efficacy and prosocial tendencies can play a mediating role between PTG and entrepreneurial intentions in the post-COVID-19 era. The results shed light on the relationship between PTG and entrepreneurial intentions in the post-COVID-19 era and may promote the application of the social cognitive theory to the studies on entrepreneurial intentions.

## Practical Contributions

The findings of this study provide some constructive and practical suggestions. First, since college students’ PTG significantly and positively affects entrepreneurial intentions in the post-COVID-19 era, colleges and universities should pay attention to their PTG, provide regular psychological counseling to the students who have experienced trauma, and provide them with interpersonal support and enable them to enjoy teachers and classmates’ company by conducting group activities. These measures can effectively increase the college students’ PTG levels.

Second, given that entrepreneurial self-efficacy partially mediates the relation between PTG and entrepreneurial intentions, universities should adopt measures to improve students’ entrepreneurial self-efficacy while providing them entrepreneurship education. These measures include focusing on entrepreneurship guidance and entrepreneurship practice when teaching courses on the entrepreneurship theory; holding up some entrepreneurs as models to bolster their entrepreneurial confidence; and improving supporting policies and measures for them to start their businesses.

Third, given that the prosocial tendency partially mediates the relation between PTG and entrepreneurial intentions, colleges should praise and encourage the students who derive pleasure from helping others by holding up them as models. Teachers should inculcate empathy and habit of thinking from others’ perspective in students through classroom activities. In addition, colleges and universities should cultivate college students’ group cooperation spirit by adopting measures such as group competitions, thereby enhancing their prosocial tendencies.

Finally, the results of this study reveal that entrepreneurial self-efficacy and prosocial tendencies have a significant chain mediating effect on the relationship between PTG and entrepreneurial intentions. Therefore, universities should intensify their efforts to enhance students’ entrepreneurial self-efficacy and prosocial tendencies to improve their level of PTG and entrepreneurial intentions. Entrepreneurial self-efficacy can in turn positively influence prosocial tendencies and thus enhance entrepreneurial intentions. Thus, the chain mediation model established in this study has a certain degree of practical contributions.

## Limitations and Future Directions

This study also has some limitations. First, we surveyed college students from only a single university in Hebei, China. Thus, future studies should aim to expand the scope of the investigation to further vindicate the findings of this study. Second, this study considered only entrepreneurial self-efficacy and prosocial tendencies as mediating variables between PTG and entrepreneurial intention. Studies are required to explore whether there are more mediating variables in the process or whether the mediating variables are moderated by other variables. Finally, this study used cross-sectional data; thus, the relationship between the variables could be confirmed at a specific time point. We aim to adopt lagged data in future studies to better understand the dynamic process of the change in the relationship between the variables.

## Data Availability Statement

The raw data supporting the conclusions of this article will be made available by the authors, without undue reservation.

## Ethics Statement

The studies involving human participants were reviewed and approved by Hengshui University. The patients/participants provided their written informed consent to participate in this study. Written informed consent was obtained from the individual(s) for the publication of any potentially identifiable images or data included in this article.

## Author Contributions

LW designed the study, analyzed the data, and drafted the manuscript. JH assisted in analyzing and interpreting the data and participated in the revision of the manuscript. All authors contributed to the study and approved the submitted version.

## Conflict of Interest

The authors declare that the research was conducted in the absence of any commercial or financial relationships that could be construed as a potential conflict of interest.

## Publisher’s Note

All claims expressed in this article are solely those of the authors and do not necessarily represent those of their affiliated organizations, or those of the publisher, the editors and the reviewers. Any product that may be evaluated in this article, or claim that may be made by its manufacturer, is not guaranteed or endorsed by the publisher.
